# The *Drosophila *IKK-related kinase (Ik2) and Spindle-F proteins are part of a complex that regulates cytoskeleton organization during oogenesis

**DOI:** 10.1186/1471-2121-9-51

**Published:** 2008-09-17

**Authors:** Dikla Dubin-Bar, Amir Bitan, Anna Bakhrat, Rotem Kaiden-Hasson, Sharon Etzion, Boaz Shaanan, Uri Abdu

**Affiliations:** 1 Department of Life Science, National Institute for Biotechnology in the Negev, Ben-Gurion University, Beer-Sheva, 84105 Israel; 2 Department of Life Science, Ben-Gurion University, Beer-Sheva, 84105 Israel; 3 National Institute for Biotechnology in the Negev, Ben-Gurion University, Beer-Sheva, 84105 Israel

## Abstract

**Background:**

IkappaB kinases (IKKs) regulate the activity of Rel/NF-kappaB transcription factors by targeting their inhibitory partner proteins, IkappaBs, for degradation. The *Drosophila *genome encodes two members of the IKK family. Whereas the first is a kinase essential for activation of the NF-kappaB pathway, the latter does not act as IkappaB kinase. Instead, recent findings indicate that Ik2 regulates F-actin assembly by mediating the function of nonapoptotic caspases via degradation of DIAP1. Also, it has been suggested that *ik2 *regulates interactions between the minus ends of the microtubules and the actin-rich cortex in the oocyte. Since *spn-F *mutants display oocyte defects similar to those of *ik2 *mutant, we decided to investigate whether Spn-F could be a direct regulatory target of Ik2.

**Results:**

We found that Ik2 binds physically to Spn-F, biomolecular interaction analysis of Spn-F and Ik2 demonstrating that both proteins bind directly and form a complex. We showed that Ik2 phosphorylates Spn-F and demonstrated that this phosphorylation does not lead to Spn-F degradation. Ik2 is localized to the anterior ring of the oocyte and to punctate structures in the nurse cells together with Spn-F protein, and both proteins are mutually required for their localization.

**Conclusion:**

We conclude that Ik2 and Spn-F form a complex, which regulates cytoskeleton organization during *Drosophila *oogenesis and in which Spn-F is the direct regulatory target for Ik2. Interestingly, Ik2 in this complex does not function as a typical IKK in that it does not direct SpnF for degradation following phosphorylation.

## Background

Protein kinases of the IκB kinase (IKK) family are known for their roles in innate immune response signaling pathways in both mammals and *Drosophila *[1-3]. All mammalian IKKs studied so far have roles in immune responses, but operate on different targets. IKKs are multi-subunit complexes consisting of two catalytic subunits (IKKα and IKKβ) and a structural component (IKKγ/NEMO). IKKα and IKKβ were identified in a protein complex that phosphorylates IκB and targets it for degradation, thereby allowing the nuclear localization and activation of NF-κB transcription factors [4-6]. The isoforms IKKε and TANK binding kinase 1 are required to for phosphrylation and activation of the transcription factor interferon regulatory factor 3 in response to viral infection [7-9]. Two members of the IKK family are known in *Drosophila*, namely *DmIKKβ *and *Ik2 *[10]. *DmIKKβ *performs similarly to the mammalian IKKα and participates in antibacterial innate immune response [11,12]. In contrast, *ik2 *(also known as *DmIKKε*) was shown to control actin and microtubule (MT) organization in an NF-κB-independent pathway [10,13,14].

Recently it was reported that Ik2 binds to *Drosophila *inhibitor of apoptosis 1 (DIAP1) and accelerates its degradation in a kinase-dependent manner [13]. One of the nonapoptotic processes that Ik2 regulates through the DIAP1/caspase pathway is assembly of the actin cytoskeleton [14]. In *ik2 *loss-of-function mutants, tracheal terminal cells, bristles, and the antenna arista laterals, all of which require accurate F-actin assembly for their polarized elongation, exhibited aberrantly branched morphology. These phenotypes were sensitive to a change in the dosage of *DIAP1 *and the caspase *DRONC *without apparent change in cell viability [13]. In addition, over-expression of Ik2 destabilized F-actin based structures. These results suggest that Ik2 may act as a negative regulator of F-actin assembly, maintaining the fidelity of polarized elongation during cell morphogenesis by modulating the level of DIAP1.

A different aspect of the *ik2 *role in cytoskeleton related processes is revealed through oogenesis studies. During oogenesis, *ik2 *is required in an NF-κB-independent process for localization of *oskar *and *gurken *mRNAs [10]. As a result, females that lack *ik2 *in the germline produce embryos that are bicaudal, ranging from headless embryos to embryos with a duplicated abdomen in place of the head and thorax. They also exhibit a ventralized phenotype. Abnormal mRNA localization in *ik2 *mutant oocytes could be attributed to defects in the organization of MT minus-ends. In addition, *ik2 *mutant oocytes and mutant escaper adults have abnormalities in the organization of the actin cytoskeleton [10]. However, the regulatory target for *ik2 *in controlling the oocyte cytoskeleton is still unknown.

In a global two-hybrid screen, Ik2 was found to interact with Spindle-F (Spn-F) (CG12114) [15]. Our previous work has shown that this newly discovered protein, Spn-F is part of a yet uncharacterized pathway leading to the organization of a distinct subset of MTs in the *Drosophila *oocyte [16]. *spn-F *was first identified as a maternal effect mutation that affects the dorsal-ventral polarity of the eggshell [17]. The asymmetric distribution of maternal determinants (*gurken*, *bicoid *and *oskar *mRNAs) in the oocyte was analyzed, and it was found that *spn-F*, like *ik2*, is required for proper localization of *gurken *during oogenesis. In addition to the maternal effect, *spn-F*, like *ik2*, also affects the bristle morphology of the adult fly. Moreover, in *spn-F *mutants, α-tubulin is abnormally associated with the oocyte nuclear periphery, and green fluorescent protein (GFP)-Tau fusion protein accumulates abnormally around the oocyte nucleus. *spn-F *was cloned and found to encode a novel coiled-coil protein, which co-localizes specifically to oocyte cortex regions, where the minus ends of MTs reside, and also occurs in a punctate granular pattern in the nurse cells [16]. Thus, earlier work show that *spn-F *affects oocyte axis determination and the organization of a subset of MTs during *Drosophila *oogenesis.

Since *ik2 *mutants share many phenotypes with *spn-F*, including a very similar bristle phenotype, ventralized eggshells, and specific effects on MT organization in oogenesis [10], we decided to study the nature of the interaction between these two proteins. We show that Ik2 directly interacts with Spn-F and that the C-terminus of Spn-F is crucial for this interaction. We also demonstrate that Ik2 is capable of phosphorylating Spn-F, but that such phosphorylation is not essential for their interaction and also does not lead to Spn-F degradation. We find that Ik2 and Spn-F co-localize to the anterior ring of the oocyte and in a punctate structure in the nurse cells. Furthermore, we show that Spn-F and Ik2 are mutually required for this localization. We therefore conclude that Ik2 and Spn-F form a complex that regulates cytoskeleton organization during *Drosophila *oogenesis. Our finding that phosphorylation of Spn-F by Ik2 has no effect on Spn-F protein degradation, contrary to the degradation seen in the case of DIAP1, demonstrates a new mode of action for Ik2 in the germline.

## Results

### Spn-F interacts physically with Ik2

**Figure 1:**
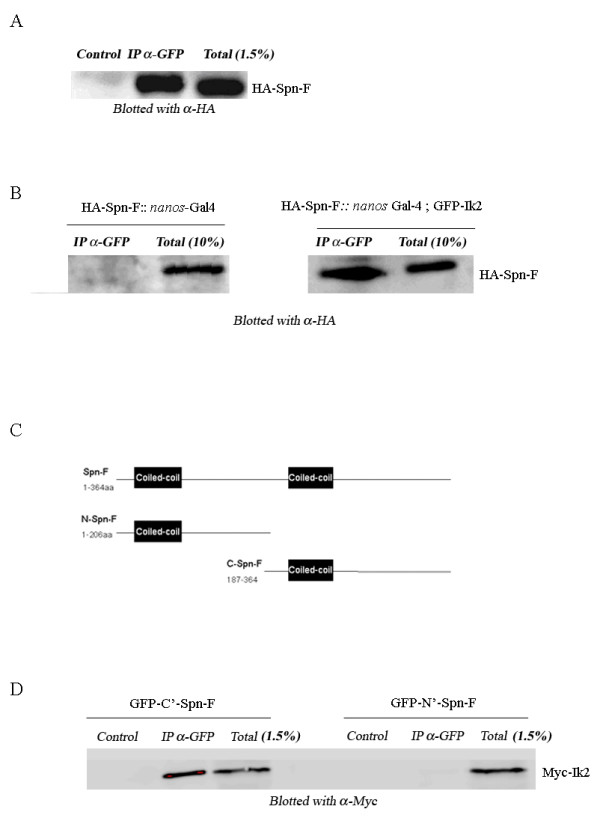
**Spn-F interacts physically with Ik2 and the C-terminus of Spn-F is crucial for this interaction**. (A) Co-immunoprecipitation of GFP-Ik2 with HA-Spn-F. Proteins were extracted from S2 cells co-transfected with GFP-Ik2 and HA-Spn-F. Untreated lysate was run on gel (Total). The same lysate was incubated with α-GFP antibody and precipitated by protein A-coated beads (IP-α-GFP), or was precipitated by protein A-coated beads without anti-GFP antibody (control). To detect Spn-F, western blotting was performed using α-HA antibodies. HA-Spn-F was successfully precipitated with GFP-Ik2. (B) Co-immunoprecipitation of GFP-Ik2 with HA-Spn-F in the germline. Proteins were extracted from transgenic fly ovaries expressing either GFP-Ik2 with HA-Spn-F (right panel) or HA-Spn-F (control, left panel) in the germline. Ovarian extracts were incubated with α-GFP antibody and precipitated by protein A-coated beads (IP-α-GFP). To detect Spn-F western blotting was performed using α-HA antibodies. HA-Spn-F was successfully precipitated with GFP-Ik2. (C) Schematic diagram of Spn-F protein coil-coiled domains and truncated protein used in D. (D) Co-immunoprecipitation assay of myc-Ik2 with GFP-N'-Spn-F or GFP-C'-Spn-F. Proteins were extracted from S2 cells co-transfected with myc-Ik2 and GFP-N'-Spn-F or GFP-C'-Spn-F. Untreated lysate was run on gel (Total). The same lysate was incubated with α-GFP antibody and precipitated by protein A-coated beads (IP-α-GFP), or was precipitated by protein A-coated beads without anti-GFP antibody (control). To detect Ik2, western blotting was performed using α-myc antibodies. Myc-Ik2 was co-precipitated with the C-terminus of Spn-F but not with the N-terminus of Spn-F.

In a yeast two-hybrid-based protein-interaction map of the fly proteome, several proteins were found to interact with Spn-F, among them Ik2 kinase [15]. To further verify the yeast two-hybrid interaction, we performed a co-immunoprecipitation (co-IP) assay in Schneider (S2) cells. Extracts from S2 cells co-expressing GFP-tagged Ik2 and hemagglutinin (HA)-tagged Spn-F were immunoprecipitated with antibodies against GFP and immunoblotted with antibodies against HA. Our results demonstrated that it is possible to co-precipitate Ik2 together with Spn-F (Fig 1A), indicating that Spn-F interacts with Ik2, in agreement with the findings of the yeast two-hybrid assay. Next we sought to establish whether Spn-F and Ik2 also interact physically in the germline. For that purpose we expressed HA::Spn-F along GFP::Ik2 in the germline using the UAS/Gal4 system. GFP::Ik2 was immunoprecipitated from ovarian extract, and the presence of Spn-F was studied by western blot with antibodies against HA (Fig. 1B). Similarly to our results in S2 cells, we were able to show that Spn-F is found in a complex with Ik2 in the oocyte as well (Fig, 1B, right panel).

### The C-terminus of Spn-F is crucial for the interaction with Ik2

Spn-F protein is predicted to have two coiled-coil domains . The first domain extends from amino acid 32 to amino acid 114 and the second from amino acid 210 to amino acid 243 (Fig. 1C). In order to determine which domain is important for the interaction with Ik2, we fused the N-terminal part of Spn-F (1–162 aa) and the C-terminal (165–364 aa) of Spn-F to GFP. We co-expressed Myc-tagged Ik2 with each of the GFP-tagged truncated proteins in S2 cells. Extracts were immunoprecipitated with anti-GFP, and immunoblotting was done using anti-Myc. Myc tagged Ik2 was co-precipitated with the C-terminus of spn-F but not with the N-terminus of Spn-F (Fig. 1D). These results suggest that the C-terminus of Spn-F is crucial for the interaction with Ik2.

### Spn-F and Ik2 are co-localized in punctate structures in S_2_R+

**Figure 2:**
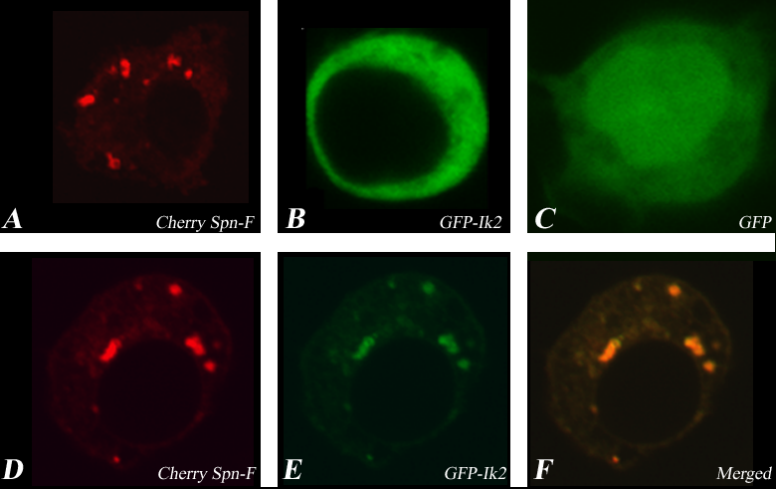
**Spn-F and Ik2 are co-localized to distinct vesicles in S_2_R+**. Confocal images of (A) Cherry-tagged Spn-F (the protein is localized to distinct vesicles in the cell cytoplasm) and (B) GFP-tagged Ik2 (the protein is evenly distributed in the cell cytoplasm). (C-E), Co-transfection of Cherry-tagged Spn-F and GFP-tagged Ik2: (C) Cherry-tagged Spn-F, (D) GFP-tagged Ik2, (E) merged picture. GFP-tagged Ik2 localization has been altered and it is now co-localized to distinct cytoplasmic vesicles with the Cherry-tagged Spn-F protein.

Having established that Spn-F interacts physically with Ik2 using co-IP in S2 cells, we decided to confirm the localization pattern of these proteins in S_2_R+ cells. Expression of Cherry-tagged Spn-F revealed that the protein is found in cytoplasmic punctate structures (Fig. 2A), whereas expression of GFP-tagged Ik2 alone showed an even distribution in the cell cytoplasm (Fig. 2B). The same localization pattern for Ik2 was reported by [14]. When we co-expressed GFP-Ik2 together with Cherry-Spn-F in S_2_R+ cells, we found that Spn-F and Ik2 are co-localized to the same cytoplasmic punctate structures (Fig 2E). These results, together with our co-IP findings, indicate that co-expression of both proteins is required for co-localization to the same vesicles in S_2_R+ cells.

### Biomolecular interaction analysis of Spn-F and Ik2 complex

**Figure 3:**
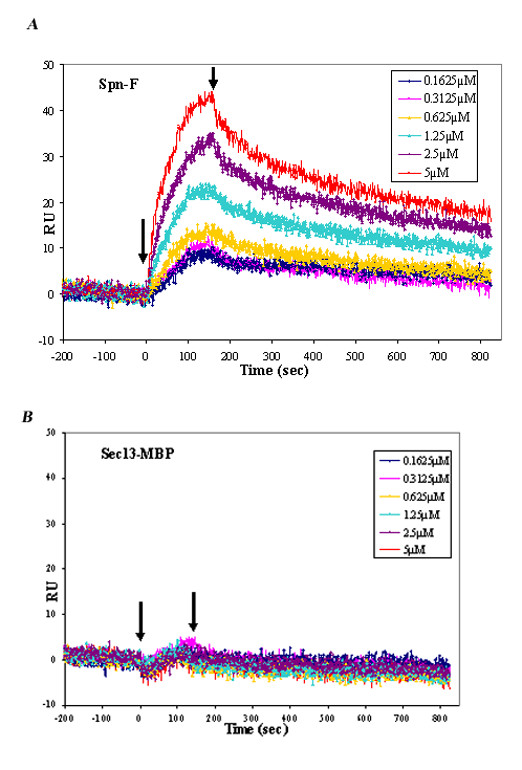
**Biomolecular interaction analysis of Spn-F and Ik2 complex**. Representative data set used for kinetic analysis of the interaction of Spn-F with Ik2-MBPHis. The figure depicts the concentration-dependent binding of Spn-F to1400 RU of captured Ik2MBPHis ligand. (A) Analyte protein injections at the indicated concentrations. (B) Graph illustrates lack of binding of Spn-F to a different MBP fused protein,. Sec13-MBP. This is a representative result from five different experiments.

To test whether the interaction between Spn-F and Ik2 protein was direct and also to enable biomolecular interaction analysis of Spn-F with Ik2, we used the multichannel ProteOn system (Bio-Rad). This method allowed us to collect kinetic data for six different concentrations of analyte at the same time [18]. Fig. 3A shows representative sensorgrams for binding of Spn-F in six concentrations to Ik2. The results revealed dose-dependent formation of this complex. In order to verify specific binding of Spn-F to Ik2 as opposed to binding to maltose binding protein (MBP), we examined the binding of Spn-F to a different MBP-fused protein, namely Sec13-MBP (Fig. 3B). These results show that there is no binding of Spn-F to Sec13-MBP, indicating that the Spn-F binding to Ik2 is specific. The data were globally fitted to the simple 1:1 interaction model with χ^2 ^of 1.63. The association and dissociation rate constants are Ka = 4.15 × 10^3 ^M^-1^.s^-1 ^and Kd = 1.69 × 10^-3 ^s^-1^, respectively. The KD equilibrium dissociation constant, which is derived from KD = kd/ka, was found to be 407.2 nM. Many examples of protein-protein interaction with affinities in the nanomolar to molar range have been described. For example, the K_D _value of complex formation between EGF and EGF receptor is 410 nM [19]. Affinity values in the range of 0.5–500 nM were observed in antibody/antigen complexes from hybridoma culture supernatants [20]. K_D _of 4.67 μM was measured between AMP-activated protein kinase (AMPK) and rat acetyl-CoA carboxylase-1 (ACC1) [21]. The affinity between Spn-F and Ik2 falls in the nanomolar range indicating a stable complex formation resembling that of antibody-antigen complexes.

### Ik2 phosphorylates Spn-F

**Figure 4:**
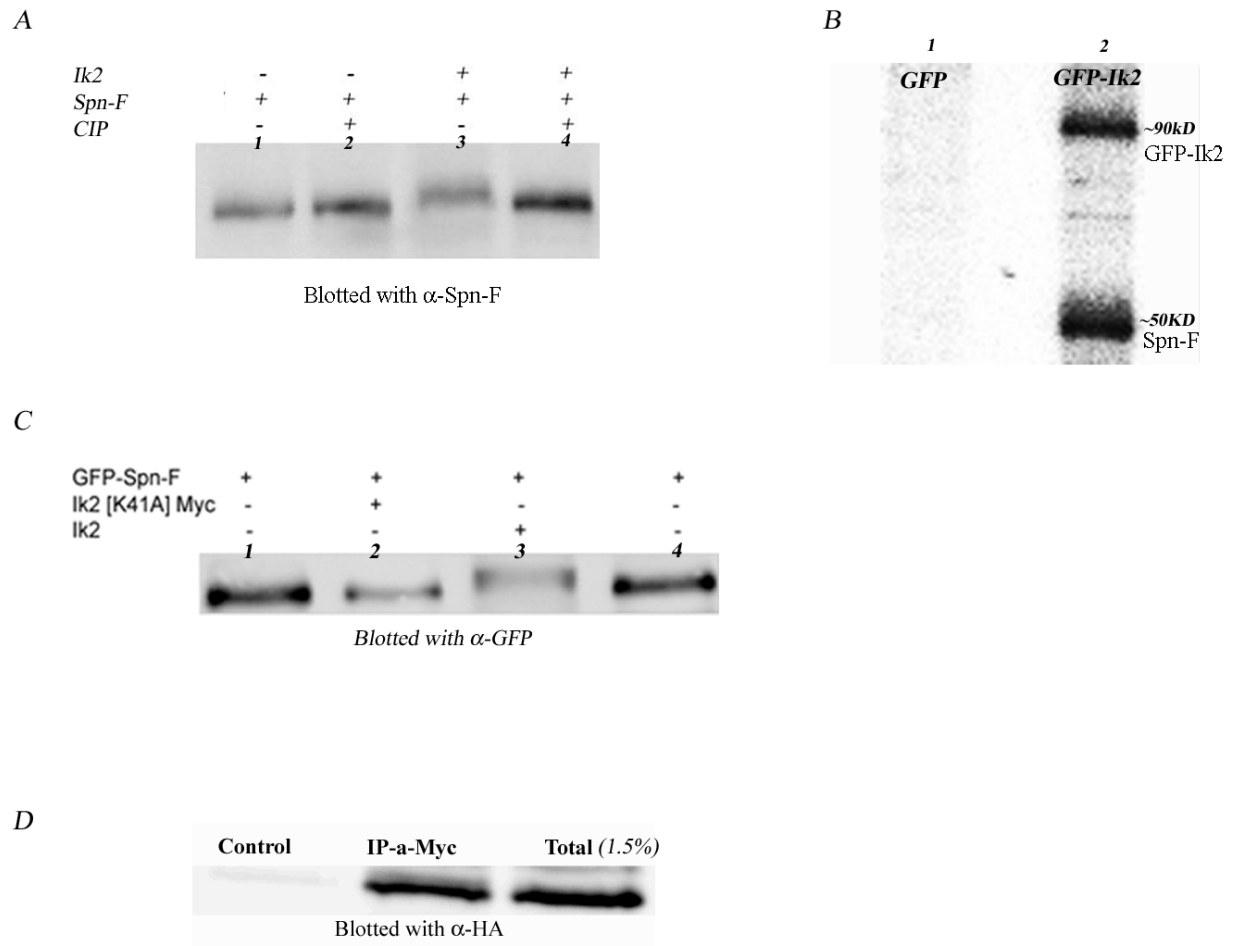
**Ik2 phosphorylates Spn-F and the phosphorylation is not essential for physical interaction between the proteins**. (A) Western blot analysis for mobility shift assay. Lane 1 – S2 cells expressing Spn-F. Lane 2 – S2 cells expressing Spn-F that were treated with calf intestinal alkaline phosphatase (CIP). Lane 3 – S2 cells co-expressing Spn-F and Ik2. Lane 4 – S2 cells co-expressing Spn-F and Ik2 that were treated with CIP. Spn-F co-expressed with Ik2 migrates more slowly on the gel than Spn-F alone or after treatment with CIP. (B) In vitro kinase assay. Autophosphorylation of Ik2 and phosphorylated Spn-F is indicated in lane 2. GFP or GFP-Ik2 was expressed in S2 cells and immunoprecipitated by anti-GFP. The immunoprecipitated proteins were subjected to an in vitro kinase assay using recombinant HIS-Spn-F protein. (C) Western blot analysis for mobility shift assay. Lanes 1, 4 – S2 cells expressing GFP-Spn-F alone. Lane 2 – GFP-Spn-F and kinase dead Ik2 – IK2 [K41A]-Myc. Lane 3 – GFP-Spn-F and Ik2. Kinase dead Ik2 does not affect the mobility of Spn-F whereas Ik2 retards its migration on the gel. (D) Co-immunoprecipitation of Ik2 [K41A]-Myc with HA-Spn-F. Proteins were extracted from S2 cells co-transfected with Ik2 [K41A]-Myc and HA-Spn-F. Untreated lysate was run on gel (Total). The same lysate was incubated with α-myc antibody and precipitated by protein A-coated beads (IP-α-myc), or was precipitated by protein A-coated beads without anti-myc antibody (control). To detect Spn-F, Western blotting was performed using α-HA antibodies. HA-Spn-F was successfully precipitated with kinase dead Ik2.

Since Ik2 is a serine/threonine kinase and binds to Spn-F protein, we investigated whether Ik2 phosphorylates Spn-F protein. S2 cells were co-transfected with Ik2 and Spn-F or Spn-F alone. Extracts were subjected to SDS-polyacrylamide gel electrophoresis (SDS-PAGE), and Spn-F protein migration on gel was analyzed by western blot with an anti-Spn-F antibody. In cells co-expressing Spn-F and Ik2, we found a reduction in the mobility of Spn-F protein (Fig 4A, lane 3) as compared with its migration when expressed alone (Fig. 4A, lane 1). This reduced mobility of Spn-F could be mitigated by treatment with calf intestinal alkaline phosphatase (Fig 4A, lane 4), strongly suggesting that Ik2 phosphorylates Spn-F. To confirm these results, we performed an in vitro phosphorylation kinase assay. GFP-tagged Ik2 was immunoprecipitated from S2 cells. We studied the ability of this immunoprecipitate to phosphorylate purified His-tagged Spn-F protein. We found that besides the autophosphorylation of GFP-Ik2 protein, there was another ^32^P-labeled protein band at ~50 kD, which corresponds to the mass of His-tagged Spn-F (Fig 4B, lane 2). Taken together with the shift in mobility, the latter result demonstrates that Ik2 phosphorylates Spn-F.

### The interaction between Spn-F and Ik2 does not depend on phosphorylation

It was recently shown that Ik2 phosphorylates and interacts with DIAP1. The interaction between DIAP1 and Ik2 was not dependent on phosphorylation [13]. Accordingly we proceeded to test the ability of Spn-F to interact with a kinase dead form of Ik2, Ik2 K41A [14]. Western blot analysis showed that the mobility of Spn-F when expressed with Ik2 K41A was similar to the migration of Spn-F when expressed alone, proving that the kinase domain of Ik2 is inactivated in the mutant (Fig 4C, lane 2). Although Spn-F was not phosphorylated by Ik2 K41A, a specific interaction between the proteins did take place (Fig 4D), indicating that the interaction between Ik2 and Spn-F proteins does not depend on phosphorylation.

### Ik2 does not promote Spn-F degradation

**Figure 5:**
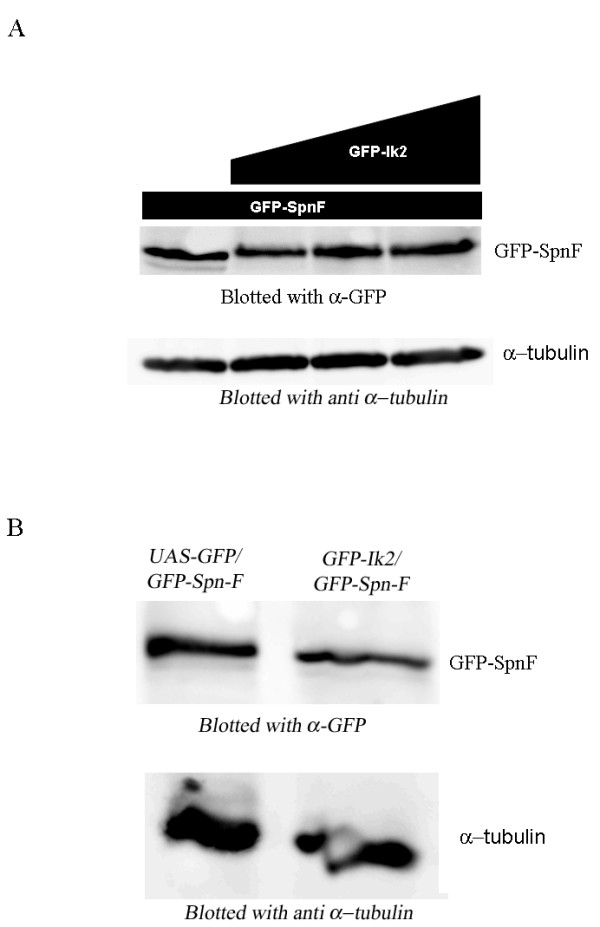
**Ik2 does not promote Spn-F degradation**. (A) Western blot analysis for Spn-F protein levels. S2 cells were co-transfected with GFP-Spn-F and increasing amounts of GFP-Ik2 (0, 200, 500,1000 ng). Ik2 did not affect Spn-F protein levels. (B) Western blot analysis for Spn-F protein stability in the germline. The level of Spn-F in the germline was examined in the presence of over-expression of Ik2. Ovarian extracts from flies expressing GFP::Spn-F and GFP::Ik2 was tested in comparison with ovarian extracts from transgenic flies expressing GFP::Spn-F or GFP. Our results showed that expression of Ik2 does not affect Spn-F stability in the germline.

It was recently reported that Ik2 promotes degradation of DIAP1 through direct phosphorylation [13]. Since Ik2 phosphorylates Spn-F in S_2_R+ cells, we examined whether such phosphorylation of Spn-F leads to its degradation. S_2_R+ cells were co-transfected with GFP-tagged Spn-F and increasing amounts of GFP-tagged Ik2. Cell extracts were subjected to western blot analysis with anti-GFP. We found that while expression of different levels of GFP-Ik2 did lead to Spn-F phosphorylation, as revealed by the change in Spn-F mobility, this phosphorylation did not lead to its degradation (Fig. 5A). To determine whether Ik2 influences the stability of Spn-F in flies, we tested the stability of Spn-F in the germline while over-expressing GFP::Ik2. For that purpose we generated transgenic flies expressing GFP::Spn-F together with GFP::Ik2, then tested the level of Spn-F protein relative to transgenic flies expressing GFP::Spn-F or GFP alone. We demonstrated that expression of Ik2 had no effect on Spn-F protein stability in the germline (Fig 5B), thus supporting our finding that Ik2 does not promote Spn-F degradation.

### Ik2 protein is co-localized with Spn-F in punctate form in the nurse cells and at the anterior ring of the oocyte

**Figure 6:**
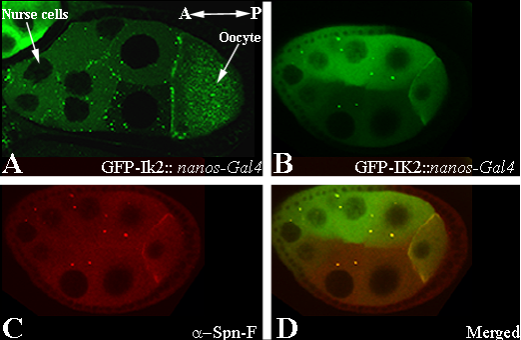
**Ik2 protein is co-localized with spn-F in the nurse cells and at the anterior ring of the oocyte**. (A) Live imaging of stage 9 egg chamber from nanos-Gal4; pUASP GFP-Ik2 transgenic flies. Ik2 is localized to the anterior end of the oocyte. (B-D) Confocal image of stage 8 egg chamber from nanos-Gal4; pUASP GFP-Ik2 transgenic flies stained with anti-Spn-F. (B) GFP-Ik2 in green; (C) immunostaining for Spn-F in red; (D) merged picture. Both proteins are co-localized along the anterior cortex of the oocyte and in a punctate pattern in the nurse cells. Egg chambers are positioned that the anterior (A) is to the left left and posterior end (P) is to the right.

In order to examine the localization of Ik2, transgenic flies expressing GFP-tagged Ik2 were generated. Expression of a GFP-tagged UASp-*ik2 *transgene under the direction of the ubiquitous *actin-Gal4 *driver rescued both the viability and bristle abnormalities of *ik2 *allelic combinations completely (data not shown). It has been reported that over-expression of Ik2 promotes DIAP1 elimination and induces cell death in somatic tissues [13]. However, we found that flies over-expressing GFP-tagged Ik2 or UAS-ik2 [10] in the germline are fertile and display no induction of apoptosis. We found that GFP-tagged Ik2 is localized to the anterior end of the oocyte and is present in a punctate pattern in the nurse cells, similarly to the pattern found for Spn-F (Fig. 6A). Next, we decided to test whether the GFP-tagged Ik2 co-localizes with endogenous Spn-F protein. For that purpose, ovaries expressing GFP-tagged Ik2 were immunostained with Spn-F antibody. We observed that Spn-F and Ik2 were co-localized at the anterior ring of the oocyte and in the same punctate structures in the nurse cells (Fig. 6B–D).

### IK2 and Spn-F are reciprocally required for localization to the anterior ring in the oocyte and to the punctate pattern in nurse cells

**Figure 7:**
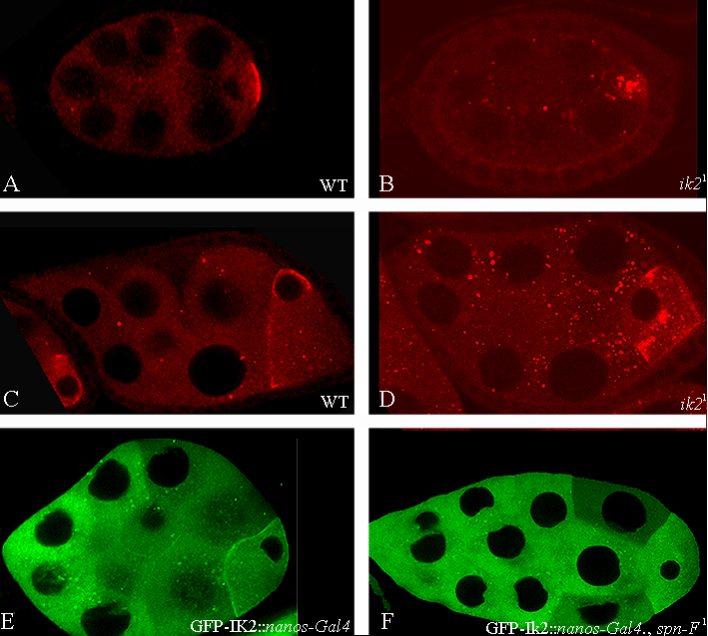
**Ik2 and Spn-F are mutually dependent for their correct localization**. (A-D) Confocal images of wild-type egg chambers (A, C) or *ik2 *germline clone egg chambers (B, D); red indicates Spn-F. In *ik2 *germ line clones during early oogenesis (B), Spn-F is no longer found in a tight posterior localization as in the wild type (A). During mid-oogenesis (D) in *ik2 *germ line clones, Spn-F localization along the anterior cortex is significantly reduced compared with the wild-type egg chamber (C) and Spn-F aggregates in the oocyte; also, there is a more punctate distribution of Spn-F protein in the nurse cells compared with the wild type (C). (E, F), Live imaging of stage 8 egg chamber from nanos-Gal4; pUASP GFP-Ik2 transgenic flies (E) or nanos-Gal4; pUASP GFP-Ik2 in *spn-F *mutant background (F). In *spn*-F mutant ovaries, Ik2 localization to the anterior ring of the oocyte is diminished and there is a reduction in the punctate form in the nurse cells.

Having shown that Ik2 and Spn-F create a complex, we tested whether the proteins are mutually required for their correct localization. To determine whether Spn-F localization depends on Ik2, we studied the localization of Spn-F in *ik2 *mutants. Because *ik2 *mutants are lethal, we used FRT/FLP recombination combined with the *ovoD *dominant female-sterile mutation to generate mutant clones in the female germline [22]. We observed that in *ik2 *germline clones Spn-F aggregates in the oocyte during early oogenesis and is no longer found in a tight posterior localization as in the wild type (Fig. 7A, B). During mid-oogenesis Spn-F localization to the MT minus end is significantly reduced in *ik2 *germline clones as compared with wild-type egg chambers, and Spn-F aggregates in the oocyte; there is also more punctate distribution of Spn-F protein in the nurse cells (Fig 7D) as compared with the wild type (Fig. 7C). We also analyzed GFP-Ik2 localization in *spn-F *mutant ovaries and found that Ik2 localization to the anterior ring of the oocyte was diminished (Fig. 7F). These results show that the proteins are mutually dependent for their correct localization.

## Discussion

Recent studies implicate the *Drosophila *IKKε homologue *ik2 *in seemingly unrelated NF-κB functions. It was shown that *ik2 *modulates caspases for a nonapoptotic function and controls both actin and MT cytoskeletons [10,13,14], and also that it regulates the actin cytoskeleton through phosphorylation and degradation of DIAP1 [14]. Moreover, it was reported that, in *ik2 *mutant oocytes, abnormal mRNA localization can be attributed to defects in organization of MT minus-ends, giving rise to ventralized and bicaudal phenotypes of mutant embryos [10]. Whereas the control of actin polymerization appears to be mediated by a nonapoptotic function of DIAP1, the regulatory target of Ik2 in controlling cytoskeleton organization in the oocyte is still unknown. In this study we examined whether *spn-F*, which showed precisely the same ovarian and bristle phenotypes as *ik2 *mutants, might be an Ik2 target. In previous work we reported that *spn-F *encodes a novel protein that affects oocyte axis determination and the organization of MTs during *Drosophila *oogenesis [16]. In this work we show that Ik2 physically interacts with Spn-F and forms a relatively stable complex. In addition, we show that Ik2 phosphorylates Spn-F but that the interaction between these two proteins is independent of phosphorylation. Thus, our results suggest that Spn-F is a putative regulatory substrate of Ik2. Moreover, our results indicate that the nature of the interaction between Spn-F and Ik2 is different from that attributed to Ik2 and DIAP1. We showed that Spn-F phosphorylation by Ik2 had no effect on its stability in S2 cells. Supporting this conclusion is the finding that over-expression of Ik2 in the ovaries had no effect on Spn-F stability or development [10; present study], indicating that over-expression of *ik2 *does not lead to Spn-F degradation. Furthermore, the ovarian phenotype of *spn-F *mutant is similar to the ovarian phenotype of *ik2 *[10,16]. Thus, whereas Ik2 regulates organization of the actin cytoskeleton via phosphorylation and degradation of DIAP1, in the oocyte, rather than affecting Spn-F degradation, Ik2 and Spn-F form a complex that regulates the oocyte cytoskeleton.

How does the IK2/Spn-F complex function in the germline? In this study, we showed that Ik2 and Spn-F are co-localized both to the anterior ring during mid-oogenesis and to punctate structures in the nurse cells. Additionally, Ik2 and Spn-F are mutually required for correct localization in the germline. In *ik2 *germline clones, Spn-F protein localization along the anterior cortex is significantly reduced relative to the wild-type egg chamber and Spn-F aggregates in the oocyte; also, there is a higher accumulation of the punctate structures containing Spn-F protein in the nurse cells as compared with the wild type. Furthermore, we found that Ik2 localization to the anterior end of the oocyte and to the punctate structure in the nurse cells depends on *spn-F*. Thus, we suggest that the correct localization of Spn-F and Ik2 complex to special compartment within the oocyte is an essential requirement for organization of oocyte cytoskeleton. The defects observed in *ik2 *and *spn-F *mutants oocyte are most likely due to misslocalization of the Ik2/Spn-F complex.

Immunostaining has shown that Spn-F protein localizes to the minus end of the MT network in the oocyte and also to granules in the nurse cells. In our previous work we found that depolymerization of MT and mutations in *Dynein heavy chain *cause a significant loss of Spn-F localization at the oocyte anterior cortex [16]. In addition, these treatments results in substantial increase in both the number and the size of Spn-F granules in the nurse cells. These observations suggest that Spn-F is transported from the nurse cells to the oocyte and that this transport could be mediated by Dynein. In the present study we found that when Ik2 was expressed alone in S2R+ cells, it was evenly distributed in the cytoplasm whereas Spn-F localized to cytoplasmic punctate structures. When both proteins were co-expressed in S2R+, Ik2 was co-localized to the cytoplasmic punctate structures along with Spn-F. Moreover, we found a higher accumulation of punctate structures containing Spn-F protein in the nurse cells in *ik2 *mutants as compared with the wild type, similarly to our observation in *Dynein heavy chain *mutant. Taking all of our results into account, we would like to propose that Spn-F is required for localization of Ik2 to cytoplasmic transport vesicles while Ik2 is required for correct transport of the complex from nurse cells to oocyte. Once the complexes are in the oocyte, they may accumulate at certain cortical sites where they promote the interaction of MTs and the actin cytoskeleton.

## Conclusion

In conclusion, we have demonstrated that Ik2 and Spn-F form a complex which regulates cytoskeleton organization during *Drosophila *oogenesis and in which Spn-F is the direct regulatory target for Ik2. Unlike other IKK proteins, Ik2 phosphorylates Spn-F without promoting its degradation.

## Methods

### Fly strains

Oregon-R and Canton-S were used as a wild-type control. The following mutant and transgenic flies were used: *spn-F*^1^, *spn-F*^2^, pUASp HA tagged *spn-F *[16], *ik2*^1 ^[10]. Germline expression was performed with *nanos*-Gal4-VP16 [23] and ubiquitous expression with *Act5C*-GAL4 (Bloomington). Germline clones for *ik2 *were generated using the ovoD-FLP technique and FRT40A (2L) [22]. To induce expression of FLP recombinase, flies were mated for 24 hours, and second instar larvae were heat shocked in a 37°C water bath for two hours on three consecutive days.

### Constructs and transgenic flies

The entire coding sequence of enhanced green fluorescent protein (EGFP) (Invitrogen) was amplified by PCR using modified primers to create a *KpnI *restriction site at the 5' end (EGFP-KPN-F-5' GGTACCATGGTGAGCAAGGGCGAGGAGC 3') and an *Xba*I site at the 3' end (EGFP *Xba *R – 5' TCTAGACTTGTACAGCTCGTCCATGCCG 3'). The resulting PCR product was digested using *Kpn*I and *XbaI *and was cloned into pBlueScript to create the EGFP pBlueScript vector. This vector was later used to clone all of the pUASp GFP-tagged constructs. The full length *spn-F *coding sequence was amplified by PCR from EST (LD01470) using modified primers to create an *Xba*I restriction site at the 5' end (5' TCTAGAATGGAGGCATCTGCTGCCAAAATC 3') and an *Not*I site at the 3' end (5' GCGGCCGCCTGGGTCAGAAGTCACC 3'). To create the N-truncated GFP-tagged form of spn-F, the coding sequence from 1–486 bp was amplified by PCR using modified primers to create an *XbaI *restriction site at the 5' end (5' TCTAGAATGGAGGCATCTGCTGCCAAAATC 3') and an *NotI *restriction site at the 3' end (5' GCGGCCGCTCATTGGCATCTGGTTCACT 3'). To create the C-truncated GFP-tagged form of spn-F, the coding sequence from 495–1095 bp was amplified by PCR using modified primers to create an *XbaI *restriction site at the 5' end (5' TCTAGAACGCAGCACTCCCCCAATCCTCACC 3') and an *NotI *restriction site at the 3' end (5' GCGGCCGCCTGGGTCAGAAGTCACC 3'). To create a full length GFP-tagged form of Ik2, the entire coding sequence of Ik2 was amplified by PCR from EST (SD10041) using modified primers to create an *Xba*I restriction site at the 5' end (5' TCTAGAATGTCCTTCCTGCGCGGTTCCGTAAGC 3') and an *Not*I site at the 3' end (5' GCGGCCGCCTAACTACTTTCCAGACTTCCG 3'). All of the above PCR products were digested using *Xba*I and *NotI *and were cloned into EGFP-pBlueScript. The resulting pBlueScript vectors were cut using *KpnI *and *NotI *and the inserts cloned into pUASp vectors. To create an mCherry-tagged Spn-F, mCherry in pCS2 was amplified by PCR, using modified primers to create *Kpn*I restriction sites at both ends (5' CGGGGTACC ATGGTGAGCAAGGGCGAGG 3' and 5' CGGGGTACC CTTGTACAGCTCGTCCATGCCGC 3'). The resulting PCR product was digested with *Kpn*I and then inserted into a pUASp vector containing Spn-F coding sequence from EST (LD-01470). P-element-mediated germline transformation of this construct was carried out according to standard protocols [24]. Ten independent lines were established from each construct.

### Antibody Staining

Ovaries were dissected in PBS, then fixed for 20 minutes (3.8% formaldehyde in PBS and Heptane) and washed 3 × 10-min in PBST (PBS, 0.3% Triton X-100). The ovaries were incubated for 1 h in PBS, 1% Triton X-100, and blocked for 1 hr in 3% BSA, PBST. After overnight incubation at 4°C with primary antibody in appropriate dilution and PBST washes, the ovaries were incubated with secondary antibody for 1 h, washed, and mounted in 50% glycerol. As a primary antibody we used monoclonal mouse anti-Spn-F (1:10; clones 8C10, 4E6 and 10C5). The secondary antibody, Cy3 goat α-mouse, was used at 1:100 (Jackson Immunoresearch). Egg chambers were imaged on a Zeiss LSM510 laser-scanning confocal microscope.

### Egg chamber live imaging

Female flies were mated and fed dry yeast for at least 3 days after eclosion. Ovaries and egg chambers were dissected in a Halocarbon oil 700 (Sigma) drop placed on a 24 × 66 mm cover glass. Confocal images were taken within 40 min after the dissection on an Olympus FV1000 laser-scanning confocal microscope.

### Cell culture and transfections

S2 or S_2_R+ cells were cultured in Schneider's cells *Drosophila *medium (Biolabs Industries, Israel) containing 10% fetal bovine serum (Biolabs industries, Israel) and penicillin (10,000 u/ml)-streptomycin (10 mg/ml)-amphotericin B (0.025 mg/ml) solution (1:100, Biolabs Industries, Israel). Cells were maintained at 25°C under normal atmosphere. 4*106 cells were transfected with 1 μg pUASp-based expression vectors and the *Act*5C-Gal4 driver by using Escort IV (Sigma). The transfected cells were cultured in Schneider's cells *Drosophila *medium without Bovine serum and antibiotics for 24 h. For western blot analysis the cells were treated in SDS sample buffer. For co-localization assay cells were fixed for 15 min (3.8% formalin in PBS), then washed 3 × 10 min in PBS and mounted in 50% glycerol. Cells were imaged on confocal microscope.

### Western blot analysis

Proteins were loaded onto a 10% polyacrylamide gel. Following electrophoresis, proteins were transferred to nitrocellulose membranes (PROTRAN, Schleicher & Schuell) for 1 h at 300 mA. The nitrocellulose membranes were blocked by incubation in TTBS (0.2 M Tris, 1.5 M NaCl, 9 mM Tween 20) containing 5% nonfat dry milk, for 30 min at room temperature followed by 1 h incubation in primary antibody. The membranes were washed in TTBS and incubated for 30 min with either a horseradish peroxidase (HRP)-labelled anti-rabbit or anti-mouse antibodies (Amersham) at a 1:2000 dilution each. The signals were visualized using ECL detection kit (biological industries). Primary antibodies used were: anti-HA (1:500, Santa Cruz Biotechnology), polyclonal anti Spn-F (1:1000), anti-GFP (1:1000, Roche diagnostic), anti-Myc (1:1000, Santa Cruz Biotechnology), anti α-tubulin (1:1000, Sigma). For phosphatase treatment, cell extracts were incubated with calf intestinal phosphatase (10 units, New England Biolabs) at 37°C for 15 min. The reaction was stopped by the addition of sample buffer.

### Co-immunoprecipitation assay

Ovaries from GFP-Ik2; HA-Spn-F/*nanos- Gal4 *or HA-Spn-F/*nanos Gal4 *expressing flies were dissected in PBS. Lysates were prepared by grinding 10 ovaries for 15 sec in 50 μl cold lysis; PB, 1% NP-40 and protease inhibitors (Sigma). The lysate was spun for 5 min in a microcentrifuge at maximum speed, and 5 μl of the supernatant was set aside for use as a whole cell lysate control. The remaining lysate was pre-cleared with protein A coated beads (Adar Biotech). After a 30 min incubation on ice, the pre-cleared lysates were incubated with anti-GFP (1:250, Roch Diagnostic) and protein A coated beads. After incubation, beads were washed in lysis buffer, resuspended in an equal volume of protein gel loading buffer, and loaded onto 10% polyacrylamide gel. To detect interaction between proteins, western blot with anti-HA antibody was performed.

Cells expressing constructs as described in the text were treated with lysis buffer (PBS, 1%Triton X-100 and protease inhibitors). Pre-cleared extracts were incubated overnight at 4°C with anti-myc (1:250, Santa Cruz) or anti-GFP (1:250, Roche Diagnostic) mouse monoclonal antibodies. Immunocomplexes were recovered by incubation with protein A coated beads (Adar Biotech) for 2 h at 4°C. To detect interaction between proteins, western blot with α-HA antibody or α-myc antibody was performed. As a negative control, the ovarian lysate was precipitated by protein A-coated beads without antibody.

### Immunocomplex Kinase Assay

Cells were treated with lysis buffer containing 20 mM Tris-HCl pH 7.5, 12 mM β-glycerophosphate, 150 mM NaCl, 5 mM EGTA, 10 mM NaF, 1% Triton X-100, 1 mM DTT, 1 mM sodium orthovanadate, 1 mM PMSF, and 1.5% aprotinin. The cell extracts were clarified by centrifugation, and the supernatants were immunoprecipitated with anti-GFP. The immunocomplexes were incubated with [γ-32P] ATP and purified His-tagged Spn-F recombinant protein [16] that was used as a substrate. The kinase assay was done in a final volume of 20 μl of a solution containing 20 mM Tris-HCl pH 7.5, 20 mM MgCl2, and 100 μM ATP. The kinase reactions were stopped by adding SDS sample buffer and analyzed by SDS-PAGE. The gel was exposed to X-ray film for 24 h and visualized by a Fuji LAS3000 image analyzer.

### Protein stability assay in S_2_R+

For studying the effect of increasing levels of Ik2 on Spn-F stability, S_2_R+ cells were transfected with different concentrations of pUASp-GFP-Ik2 vector (0, 200, 500, 1000 ng) and pUASp-GFP-Spn-F (1000 ng). The expressions of the proteins were driven by Actin-GAL4 (1000 ng). Empty pUASp vector was added to adjust the total amount of expression plasmid to 3000 ng/well. Cell extracts were subjected to western blot analysis with anti-GFP antibody.

### Protein Expression and Purification

The open reading frame encoding Spn-F from EST (LD01470 was amplified by PCR using modified primers to create an *EcoRI *restriction site at the 5' end (5' CGAATTCATGGAGGCATCTGCCTG 3') and *HindIII *restriction site at the 3' end (5' CGTCAAGCTTTCAGAAGTCACCCAC 3'). The open reading frame encoding Ik2 from EST (SD10041) was amplified by PCR using modified primers to create a *BamHI *restriction site at the 5' end (5' CGGATCCCATGTCCTTCCTGCGCGG 3') and an *SalI *restriction site at the 3' end(5' GCCGTCGACCTAACTACTTTCCAGACTTC 3'). The PCR products were digested with the appropriate restriction enzymes and cloned into pMBPHis-Parallel1 [25] vector, containing N terminal MBP-His6 tags followed by a TEV proteolysis site. The plasmids coding for the fusion proteins were overexpressed in BL21 DE3 *Escherichia coli *cell, grown in 2.4l 2xYT media (containing 200 mg ampicillin). The culture was grown at 37°C to an approximate A600 O.D. of 0.4–0.6. Protein expression was induced by addition of 0.4 mM IPTG (Bio-Labs, USA), and the culture was further incubated for 2 h at 37°C. Cells were harvested at 4°C by centrifugation at 12,000 rpm. The cell pellets were resuspended in lysis buffer (Tris-HCl 20 mM pH 8, NaCl 200 mM, EDTA 1 mM, β-mercaptoethanol 5 MM, sudium azide 0.02%) containing protease inhibitors PMSF (1 mM) and E64 (10 μM) (Sigma, USA). Cells were lysed by sonication (Sonics & Materials, USA). The cell lysates were centrifuged at 12,000 rpm for 25 min. at 4°C. The supernatant solution was loaded onto an Amylose High Flow (New England bio-labs, England) column pre-equilibrated with loading buffer (Tris-HCl 20 mM pH 8, NaCl 200 mM, EDTA 1 mM, β-mercaptoethanol 5 mM,. The protein was eluted with the same buffer with the addition of 10 mM maltose. The fusion protein Ik2-MBPHis was dialysed against loading buffer containing NaCl 50 mM and eluted from an ion exchange HiTrap ANX FF column (Amersham, SwedenS) in a salt gradient of 0.05–1 M NaCl. Ik2-MBPHis was obtained in the unbound fraction. Spn-F fusion protein was further cleaved with TEV (tobacco etch virus) protease in cleavage buffer (Tris-HCl 0.5 M pH 8, EDTA 5 mM, DDT 1 mM) at 4°C overnight. The cleaved Spn-F was dialysed against loading buffer (Tris-HCl 20 mM pH 8, NaCl 200 mM, β-mercaptoethanol 5 mM, sodium azide 0.002%), and loaded onto a nickel HiTrap IMAC FF column (GEHealthcare, USA). The unbound Spn-F was dialysed against loading buffer containing NaCl 50 mM and eluted from an ion exchange HiTrap ANX FF column (Amersham, Sweden) in a salt gradient of 0.05–1 M NaCl. To verify the complete removal of MBPHis tag from Spn-F sample, western analysis with an anti-His antibody was performed; no signal was detected. Furthermore, MALDI-TOF analysis confirmed that the sample contained only pure Spn-F. Finally, the proteins Spn-F and Ik2-MBPHis were dialysed against buffer PBS.

### Binding measurements

Binding affinities of Spn-F toward Ik2 were measured using the ProteOn XPR36 Protein Interaction Array system (Bio-Rad, USA) based on surface plasmon resonance (SPR) technology [18,26]. As negative control we used Sec13-MBP protein (Dictybase ID-DDB0235182). A solution of 0.005% Tween 20 in PBS, pH 7.4, was used as running buffer at a flow rate of 30 μl/min, at 25°C. Three channels (GLC sensor chip) were activated for 5 min with a mixture of 0.2 M EDC (1-ethyl-3-(3-dimethylaminopropyl)carbodimide hydrochloride) and 0.05 M sulfo-NHS (solfo-*N*-hydroxysuccinimide). Immediately after activation of the surfaces, Ik2-MBPHis (1 μg in 10 mM sodium acetate, pH 5.5) and Sec13-MBP (1 μg in 10 mM sodium acetate, pH 4.5) were injected across channel 2 and 3, respectively. This resulted in Ik2-MBPHis coupled at response levels of 1400 RU and Sec13-MBP coupled at response levels of 5500 RU. Channel 1 served as reference. Finally, all 3 channels were blocked with 1 M ethanolamine-HCl (pH 8.5). Spn-F was then injected perpendicular to ligands, at six different concentrations, using a twofold dilution series within a range of 0.1625 to 5 μM. The six concentrations were injected simultaneously at a flow rate of 65 ul/min for 2.5 min of association phase followed by 10 min of dissociation phase at 25°C. The GLC sensor chip was regenerated with short injection of 50 mM NaOH between consecutive measurements. Results are expressed in arbitrary resonance units (RU) with subtraction of RU values from channel 1. Data were analyzed with ProteOn managerTM software, using the Langmuir model (A + B ↔ AB) for fitting kinetic data [23].

## Abbreviations

IKK: IκB kinase; DIAP1: *Drosophila *inhibitor of apoptosis 1; GFP: green fluorescent protein; MT: microtubule; MBP: maltose binding protein; co-IP: co-immunoprecipitation, HA: hemagglutinin; AMPK: AMP-activated protein kinase; ACC1: rat acetyl-CoA carboxylase-1.

## Authors' contributions

DDB and AB performed cell culture assays, confocal microscopy analysis, and transgenic flies work. AB designed and performed the immunocomplex kinase assay. RKH designed and performed the biomolecular interaction analysis. SE and BS participated in the biomolecular interaction work and analysis. UA drafted the manuscript with input from all authors. All authors read and approved the final manuscript.
